# Statistical study of ductility-dip cracking induced plastic deformation in polycrystalline laser 3D printed Ni-based superalloy

**DOI:** 10.1038/s41598-017-03051-x

**Published:** 2017-06-06

**Authors:** Dan Qian, Jiawei Xue, Anfeng Zhang, Yao Li, Nobumichi Tamura, Zhongxiao Song, Kai Chen

**Affiliations:** 1 State Key Laboratory for Mechanical Behavior of Materials, Xi’an Jiaotong University, Xi’an, Shaanxi 710049 P.R. China; 2 State Key Laboratory for Manufacturing Systems Engineering, Xi’an Jiaotong University, Xi’an, Shaanxi 710049 P.R. China; 3 Advanced Light Source, Lawrence Berkeley National Laboratory, Berkeley, California 94720 USA

## Abstract

Ductility-dip cracking in Ni-based superalloy, resulting from heat treatment, is known to cause disastrous failure, but its mechanism is still not completely clear. A statistical study of the cracking behavior as a function of crystal orientation in a laser 3D-printed DL125L Ni-based superalloy polycrystal is investigated here using the synchrotron X-ray microdiffraction. The dislocation slip system in each of the forty crystal grains adjacent to the 300 μm long crack has been analyzed through Laue diffraction peak shapes. In all these grains, edge-type geometrically necessary dislocations (GNDs) dominate, and their dislocation line directions are almost parallel to the crack plane. Based on Schmid’s law, the equivalent uniaxial tensile force direction is revealed normal to the trace of the crack. A qualitative mechanism is thus proposed. Thermal tensile stress perpendicular to the laser scanning direction is elevated due to a significant temperature gradient, and thus locations in the materials where the thermal stress exceeds the yield stress undergo plastic deformation mediated by GND activations. As the dislocations slip inside the crystal grains and pile up at the grain boundaries, local strain/stress keeps increasing, until the materials in these regions fail to sustain further deformation, leading to voids formation and cracks propagation.

## Introduction

Ductility-dip cracking (DDC) usually occurs along grain boundaries in materials with severe ductility drop in medium homologous temperature range between 0.5 and 0.8^1, 2^. It has been a long-standing nuisance for various structural materials that experience thermal treatment. Considerable efforts have been undertaken to investigate the welding induced DDC in Ni-based superalloys, in the reheated filler metals or in the heat affected zones (HAZs) of the base metals^3, 4^, under the influence of a wide range of effects such as chemical composition, element segregation, precipitate formation, crystal orientation, welding parameters, grain boundary sliding (GBS)^5–9^. It is generally accepted that DDC occurs when the accumulated strain at grain boundaries (GBs) exceeds the inherent ductility of the material and it has been proposed that dislocations are readily blocked and piled up at high-angle grain boundaries (HAGBs) although dislocation motion is not closely associated with cracking yet^10^. Our knowledge on DDC has been advanced since the development of the so-called strain-to-fracture testing approach, with which the DDC susceptibility in materials can be quantified^11, 12^. In these studies, attention has been focused on the ductility-dip temperature range and the threshold strain (ε_min_)^13^ for crack nucleation and growth, but the results reported in the literature are often contradictory and it is hard to distinguish DDC from other high temperature cracking modes, such as liquation cracking^14^. To overcome the shortcomings, an *in situ* high temperature strain testing has been conducted recently in a scanning electron microscope (SEM) to observe the DDC with submicron spatial resolution^15^. With this method, two-dimensional (2D) strain distribution is mapped pixel-by-pixel on the SEM images using digital image correlation (DIC). However, as stated in the reference paper, the computer program cannot differentiate pure strain from slippage deformation. Moreover, microstructural evolution such as defects generation and motion is difficult to characterize with SEM, so it is still unclear about how the dislocations interact with HAGBs and how the stress induced GBS is linked to cracking.

To unveil the basic mechanism, metallurgical microstructure of the metallic alloy materials needs to be investigated at the sub-granular level. At present, the most popular characterization techniques that satisfy such requirements include transmission electron microscopy (TEM), electron backscattering diffraction (EBSD) and synchrotron polychromatic X-ray microdiffraction (μXRD). Among these, TEM provides an unprecedented spatial resolution and is powerful to visualize microstructural defects such as dislocations. However, TEM sample preparation is effort consuming, and the sampling volume, as a trade-off to spatial resolution, is rather limited, and thus it is hard to cover the entire cracks, which are typically several microns long. EBSD offers submicron spatial resolution and sampling area is large enough for crack study, but requires a critical mirror-like sample surface, and is difficult to use for quantifying dislocation density distribution^16^. The lesser known μXRD technique has become more popular with the development of synchrotron facilities^17, 18^. At present, a 1 μm spatial resolution is routinely obtained at most dedicated μXRD beamlines, which is sufficient to study the DDC in Ni-based superalloys at sub-granular level. Taking advantage of the high crystal orientation resolution (0.01° or better)^19, 20^, local defect induced lattice distortion can be precisely measured, and thus plastic deformation studied. Moreover, due to the penetration of high energy synchrotron X-rays, μXRD sample preparation is kept to a minimum, and cracks can be detected even when below the sample surface^21^. However, μXRD applications are limited by the relatively low accessibility of synchrotron light sources, raising an experimental challenge: how to efficiently link the DDC with HAGBs in a statistically meaningful way, when the number of possible measurements is finite due to the rather limited experimental time?

Laser direct forming (LDF), also colloquially known as laser 3D printing, is reported to be analogous to welding and frequently produce DDC when the weight content of Al and Ti is greater than 6% in the cladding Ni-based superalloys, in both the columnar dendritic region^22, 23^ and the stray grain region^24^. With the as-deposited fine polycrystalline structure in the stray grain region^25, 26^, a crack with suitable length allows collecting diffraction patterns at sub-granular scale and in the meanwhile covering the entire crack within a reasonable period of beamtime. Hence, in this study, a 300 μm long intergranular crack in the stray grain region of Ni-based DZ125L superalloy is selected for μXRD scan. The crystal grain size ranges from 5 μm to 20 μm in diameter. The dislocation slip system in each crystal grain right next to the crack is studied by analyzing the Laue diffraction peak shapes, and then the equivalent uniaxial tensile force direction is estimated from Schmid’s law. Statistics over forty grains in the ductility dip region shows strong correlation between the dislocation line directions and the crack propagation direction, and thus sheds light on understanding the pile-up of dislocations and cracking formation mechanism at sub-granular scale.

## Results

**Figure 1:**
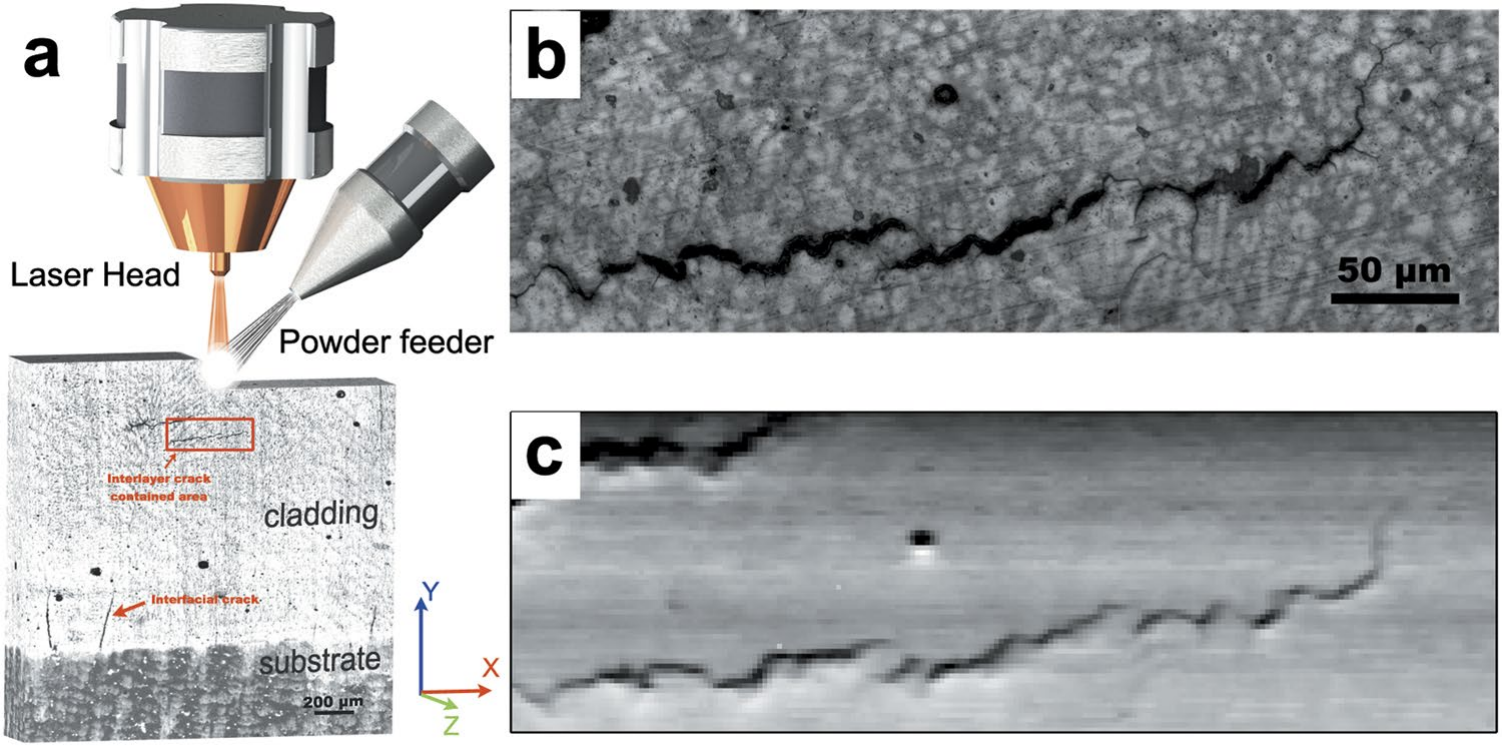
Schematic of 3D printing specimen preparation and microstructure characterization. (**a**) Laser 3D printing setup and a low magnification optical micrograph showing two types of cracks parallel and perpendicular to the substrate/cladding interface. (**b**) Optical microscope image of the investigated crack containing region. (**c**) Raw intensity map generated from μXRD to show the cracks as low intensity pixels with reasonable contrast and spatial resolution.

Schematically shown in Fig. 1a, a ~0.8 mm thick thin-walled sample was prepared by laser 3D printing without strict process optimization on a 001 directionally solidified DZ125L Ni-based superalloy. For convenience, a Cartesian coordinate system **O-XYZ** was established, with **X**-axis parallel to the laser scanning direction and **Y**-axis perpendicular to the cladding-substrate interface. In 3D printing, the surface of the substrate or the last solidified layer and the feeding powder are heated above the melting point by the focused laser to form a molten pool, generating a temperature gradient from the molten pool to the other parts of the material, especially along the Y-direction in this thin-walled structures^27^. As the laser spot is moved away, the molten material solidifies rapidly. Unintended cracks were observed under an optical microscope (OM) in the deposited materials without any post heat treatment or external mechanical load. These cracks could be clearly cataloged into two types. Some cracks, which formed upon solidification, were found close to and perpendicular to the substrate-cladding interface on the cladding side, as shown in Figure S1. From previous studies, it is known that giant columnar dendritic grains existed in this region, and the crack propagated along a HAGB. Next to the crack, only a few (generally two) crystal grains could be detected, and thus this type of crack is not suitable for a statistical study of grain orientation dependence. The other type of cracks, which formed due to thermal gradient stresses in the already solidified products, were lying farther from the substrate in the stray grain region and roughly parallel to the **X**-axis. Figure 1b shows typical cracks of this type, one lying at the top left corner of the micrograph, the other in the middle, measuring approximately 300 μm in length with a continuous zig-zagging microscopic propagation path. Short branches were also seen at several triple junctions along this crack. In this study, we will ignore the top-left one and focus on the one in the middle.

### μXRD microstructure characterization

A 400 μm by 130 μm area, almost identical to the one shown in the optical micrograph in Fig. 1b, was scanned using a micro-focused synchrotron polychromatic X-ray beam with a 2 μm step size. In order to analyze the materials microstructure and its deformation mechanism, it is essential to visualize the crack, i.e. to know where the crack was relative to the X-ray beam position within the scanned area. Hence a newly developed intensity mapping approach was employed^21^. From the generated raw intensity map (Fig. 1c), the crack appears with reasonable contrast and spatial resolution, as low intensity pixels. Therefore in our following analysis, a threshold intensity value was applied, below which the diffraction patterns would be considered to originate from deeper regions within the crack.

**Figure 2:**
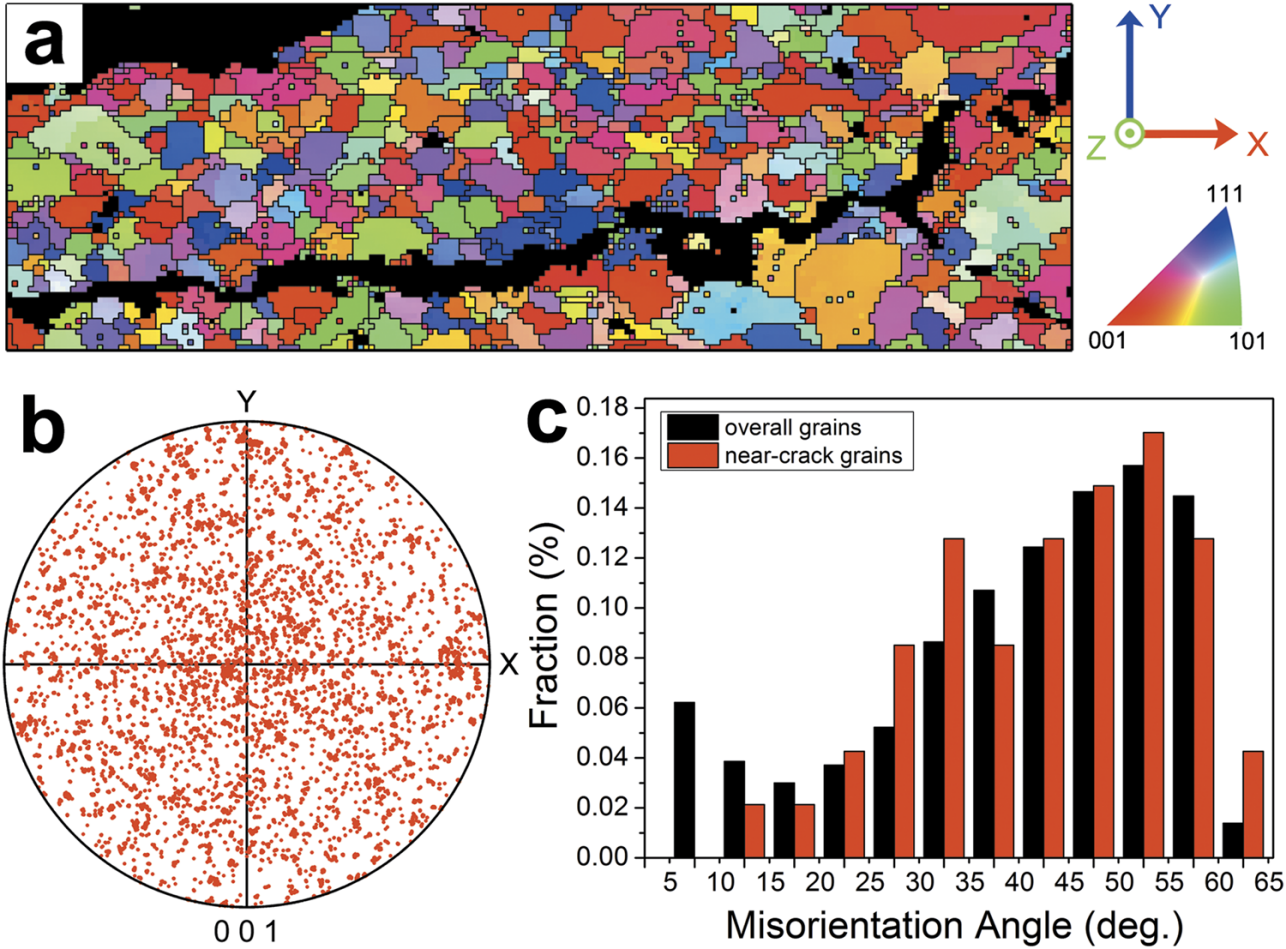
Crystal orientation characterization of the crack containing region. (**a**) The inverse pole figure map showing the crystal orientation along xx-axis. (**b**) The inverse pole figure showing random crystal orientation distribution in the scanned region. (**c**) Statistical distribution of GB misorientation for the overall scanned area and the grains adjacent to the crack, respectively.

The recorded Laue diffraction patterns were indexed using the software package XMAS^28^, and the crystal orientation distribution was mapped in the inverse pole figure of **X**-axis in Fig. 2a. The crystal grain size spanned from about 5 μm to 20 μm, and no obvious correlation could be found between grain size and distance of the grain to the crack. No deformation twinning was detected in the region investigated. From the inverse pole figure shown in Fig. 2b, it can be seen that the crystal orientation distribution in the scanned region was almost random, making this crack ideal for a statistical study of how the plastic deformation occurred during the cracking in grains in function of grain orientation. In Fig. 2c, it can be seen that the GB misorientation of the scanned area distributed mainly between 40° and 60°. By computing the misorientation angle between each pair of grains on top and at the bottom of the crack trace (the intersection of the crack plane with the cross section surface)^29^, it is confirmed that the crack is intergranular and occurred along HAGBs. The distribution of the misorientation angles along the crack is slightly higher than that of the overall map. It should be noted that it is difficult to deduce from this map the nucleation spot and propagation direction of the crack, and no clear precipitate density gradient was observed along the crack.

### 3D morphology characterization

**Figure 3:**
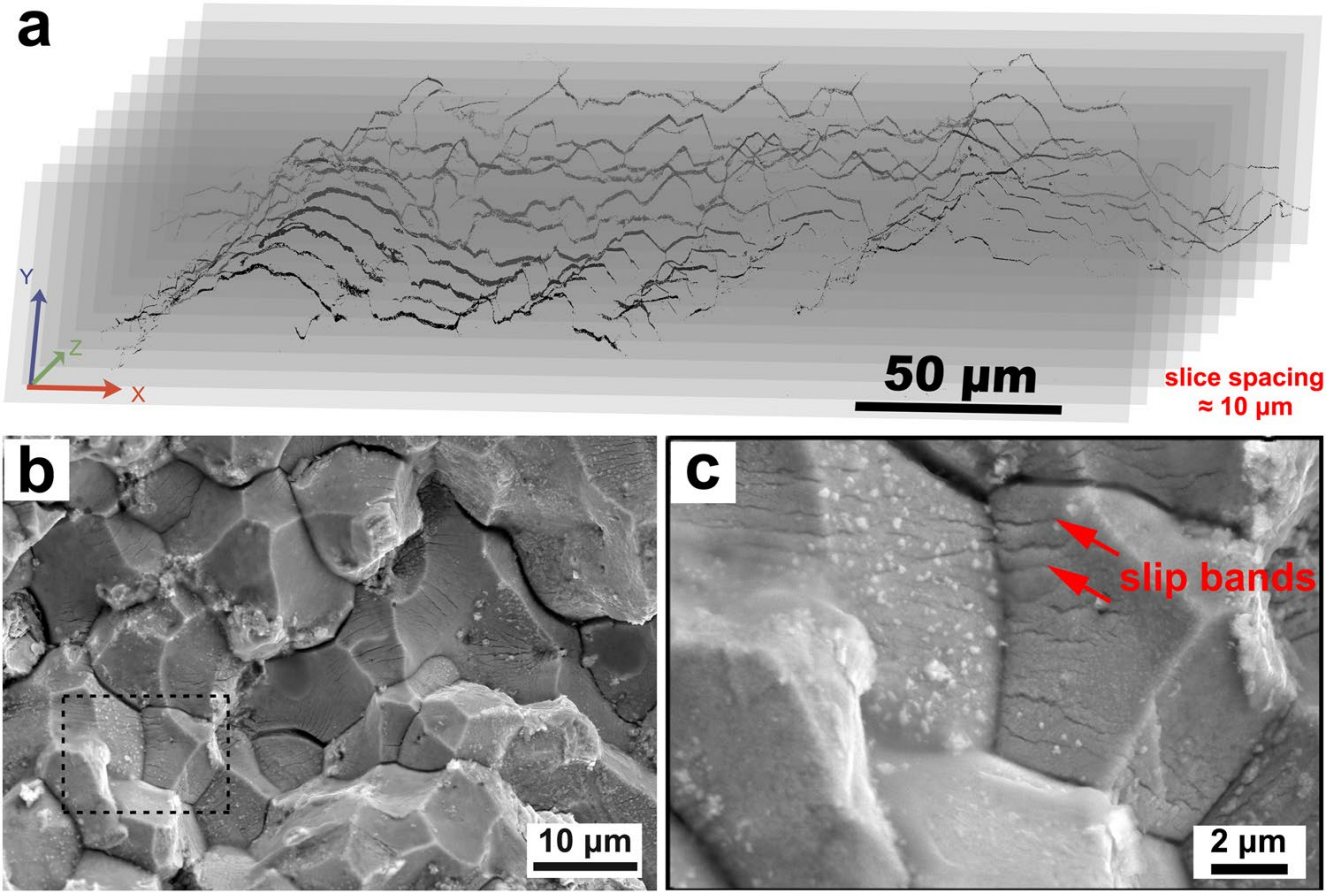
3D morphological reconstruction of the crack plane and its SEM characterization. (**a**) Slices reconstruction of the crack with the slice-spacing of ~10 μm. (**b**) Low magnification image of the crack plane, with sharp crystal facets. (**c**) The high magnification topography of the dashed area (**b**), exhibiting nano sized precipitates and slip bands (red arrows).

The 3D morphology of a crack which also appeared roughly parallel with the **X**-axis in the stray grain region was rendered with a sequential slicing method. First of all, a mirror finish of the **XY** cross-sectional region that contained a visible crack was obtained using mechanical polishing, and then the surface topology was recorded in an SEM. Then the surface was further polished off by 10 μm with a micrometer-based thickness monitoring system, before the second SEM image was taken. This procedure was repeated several times until the crack disappeared. With this method, the crack was visualized to be a corrugated plane approximately parallel to the **XZ** plane and perpendicular to the thermal gradient **Y**-direction (Fig. 3a).

### Crack plane characterization

Another crack-containing sample with the same parameters was torn off along a crack for morphological characterization of the cracking plane. In the SEM image taken in secondary electron mode (Fig. 3b), the crystal grain facets confirm, once again, that the crack propagated along GBs rather than through crystal grains, agreeing well with the cross-sectional view. The magnified image in Fig. 3c revealed a large quantity of nano-sized precipitates. Due to their tiny size and small volume fraction over the total materials, diffraction signals from the precipitates appeared too weak for phase identification, while previous literatures suggested that they were probably carbides^19^. Persistent slip bands (PSBs) were visible in most facets of the crystal grains, marked by red arrows, indicating that the high density of dislocations in the grains next to the crack must have interacted actively with the grain boundaries and crack.

### Dislocation slip system characterization

**Figure 4:**
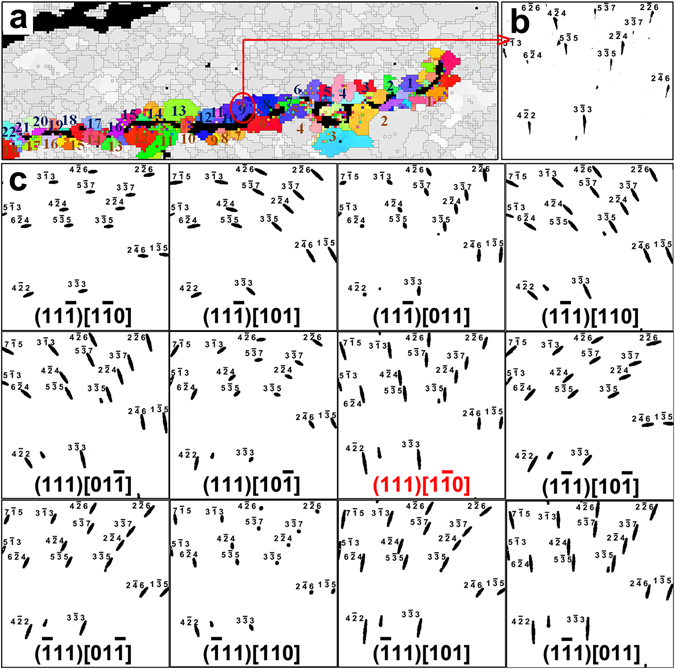
Deformation characterization of the crystal grains adjacent to the crack. (**a**) Numbered grains next to the crack, selected for plastic deformation study. (**b**) The Laue diffraction pattern with all reflections indexed as an example taken from Grain #9 on top of the crack. (**c**) Simulations of the Laue peak streaking direction corresponding to all the 12 possible {111}/〈110〉 slip systems of face-centered cubic structure by utilizing the software package XMAS. The one highlighted in red is the best match of the simulated shapes with pattern shown in (**b**).

**Figure 5:**
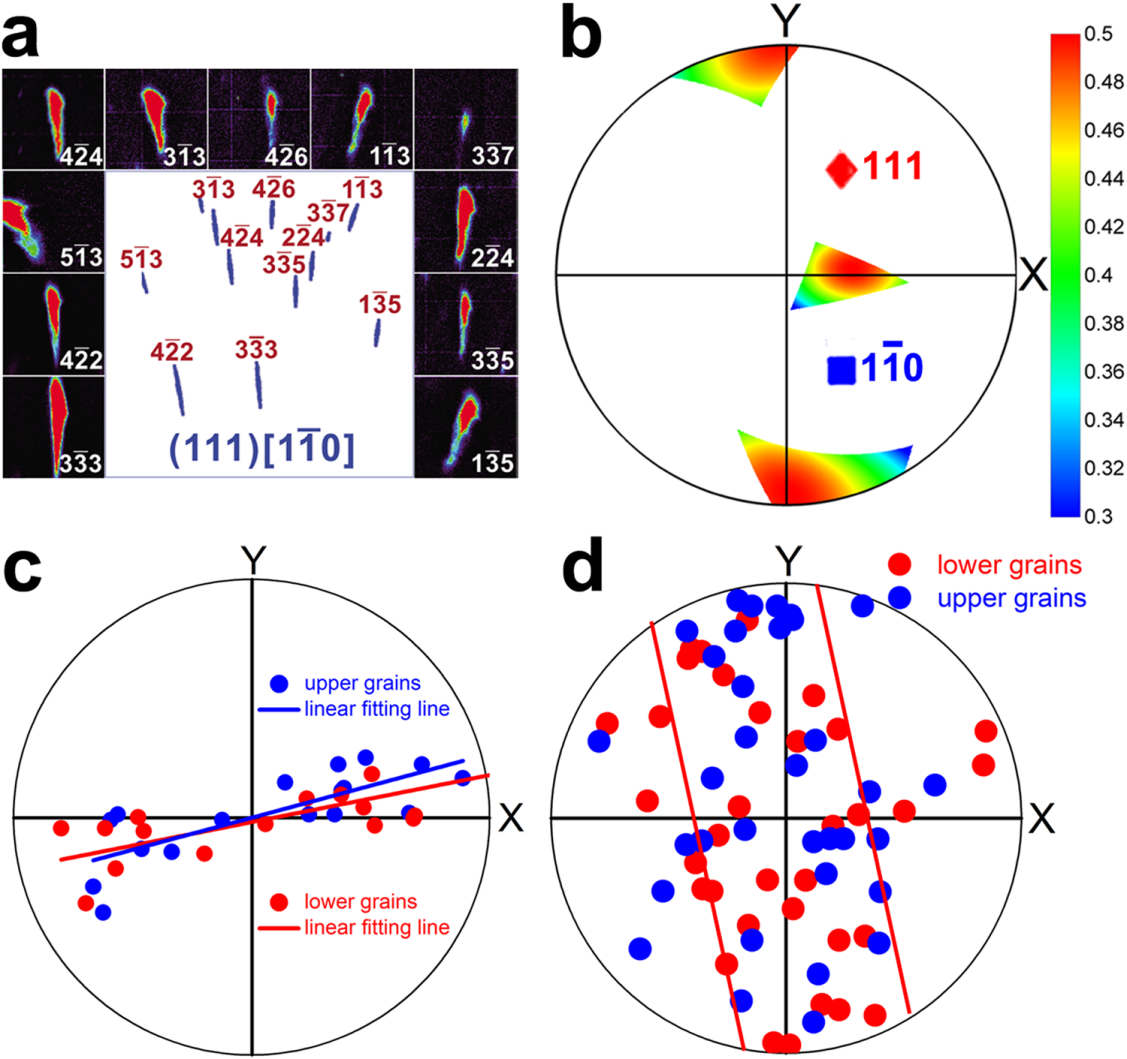
Statistical study of the dislocation slip systems of the crystal grains adjacent to the crack. (**a**) Dislocation slip system study for Grain #9. The simulated streaking Laue peaks with (111)/[1–10] GNDs are displayed surrounding the experimental observed Laue diffraction pattern. (**b**) The calculated Schmid factor as a function of equivalent uni-axial tensile stress direction. (**c**) Stereographic projection on **XY** plane of the dislocation line directions in investigated grains above (blue) and below (red) the crack, respectively. (**d**) Stereographic projection of the equivalent uni-axial tensile stress direction in each of the forty grains calculated according to Schmid’s law.

Dislocation activities were characterized for investigation of the plastic deformation occurring during cracking. A total of 40 crystal grains adjacent to the crack were selected for statistical analysis, 22 of which on the upper side and 18 on the lower side. These grains are numbered and highlighted in color in Fig. 4a. Three to five diffraction patterns in each grain were investigated, and it was concluded that the diffraction peaks were anisotropically streaked in all of them, indicating that high density of geometrically necessary dislocations (GNDs) were activated in all grains along the crack^19^. Moreover, different Laue patterns recorded in the same grain show almost identical peak shape, suggesting that the dislocation slip system was uniform in each grain. To determine the dislocation type and slip system active in each grain, experimental data were compared to simulated ones. As an example, the Laue diffraction pattern of Grain #9 with all reflections indexed is shown in Fig. 4b. The Laue peak streaking direction was simulated for each different dislocation types (edge and screw) and all the 12 possible {111}/〈110〉 slip systems of a face-centered cubic structure utilizing the software package XMAS (Fig. 4c). The simulations were based on the basic principle that different dislocation types and slip systems would induce anisotropic lattice distortion and crystal plane bending, and thus the Bragg condition will deviate from the ideal defect-free case. The simulated pattern that best match the experimental one was chosen to represent the actual active slip system. In the case of Grain #9, it is determined that edge-type (111)/[1–10] GNDs were activated. The best match simulation result for Grain #9 is shown in Fig. 5a, and the streaking Laue peaks were magnified and displayed around the simulated pattern for comparison. Once the activated slip system was determined, the direction of the uniaxial stress was derived by varying the stress direction all around the three-dimensional (3D) space and calculating the Schmid factor for each particular stress direction. According to Schmid’s law, the most probable stress direction corresponds to the highest Schmid factor, which means we can use the Schmid factor to find the highest resolved stress for the confirmed slip system and infer the tensile axis direction. As demonstrated in Fig. 5b, the direction of the uniaxial stress imposed on upper Grain #9 was, probably, along approximately either along **Y**- or **Z**.

The activated dislocation slip systems in all the selected forty crystal grains were characterized, and it was found that in all these grains the GNDs were edge-type dislocations. The dislocation line direction in each grain was projected onto the **XY** plane and plotted in Fig. 5c. Each blue dot in the figure represent the dislocation line direction in a grain on top of the crack, while the red dots represent those from the grains at the bottom. Interestingly, the blue and red points fall into the same line that approximately coincide with the average crack plane, indicating that the dislocation line direction in each individual grain was almost parallel to the crack plane according to the nearly horizontal 3D morphology of the cracking. Considering the characteristic relationship in edge dislocations, the slip planes and slip directions (Burgers vectors) in all these grains appeared normal to the dislocation trace. Similarly, the uniaxial force directions were also plotted in a stereographic projection. For most of the crystal grains investigated, the force direction projection distributed within the range indicated by the red lines in Fig. 5d.

## Discussion

Today, the microstructural mechanism of DDC is still under debate. Popular suggestions include the “creep-like” behavior and the carbide-induced tension. Lippold and co-workers (Mechanism 1) believe DDC is formed in the temperature range high enough to accommodate GBS but not high enough for dynamic recrystallization, which is analogous to the creep behavior of metals and metallic alloys^1^. GBS-induced stress concentration results in void formation at the triple junctions of GBs, while the carbide particles at the GBs reduce the stress concentration, and thus lock or resist the growth of cracks. On the other hand, Young *et al*. (Mechanism 2) suggests that the formation of intergranular carbide particles imposes a tensile stress onto the surrounding grains, and thus generates voids at GBs^8^. In both mechanisms, the mechanical deformation of the crystal grains is the prerequisite for the formation of cracks, but experimental evidence is inadequate to determine the origin of the stress.

According to the statistical study in our investigation, the uniaxial tensile stress is aligned normal to the average plane of the crack in the polycrystalline region of the deposited materials. From the fact that the laser power is distributed as a Gaussian function and from the characteristics of the laser single pass cladding process, steep temperature gradients is expected radiating from the center of the melt pool towards the solidified materials, with the temperature decreasing rapidly and vertically along the **Y**-axis from the top surface to the layers below in the **YZ** plane. The gradient is therefore perpendicular to the laser scanning direction^30^. Based on the fact that the uniaxial tension imposed on the materials is roughly parallel with the temperature gradient direction, it can be deduced that crack formation is closely related to thermal stress. The uniaxial force direction deduced from Schmid’s law is found to distribute in a range of about ±30° off the temperature gradient direction. Because the misorientation angle between a pair of {111} slip planes of FCC metallic alloys is 70.5°, we propose that the scattering of the deduced force direction mainly results from the finite number of dislocation slip systems. Although the phase, size, orientation, density, and distribution of the small sized precipitates at GBs, as well as the grain-grain interaction and grain boundary sliding can also make the force direction deviate from the thermal gradient direction, these contributions must have been minor compared to thermal stress, considering the alignment of the tensile force direction and temperature gradient. As a result, the principal driving force of the mechanical deformation and crack formation is the thermal stress for the sample investigated in this study.

**Figure 6:**
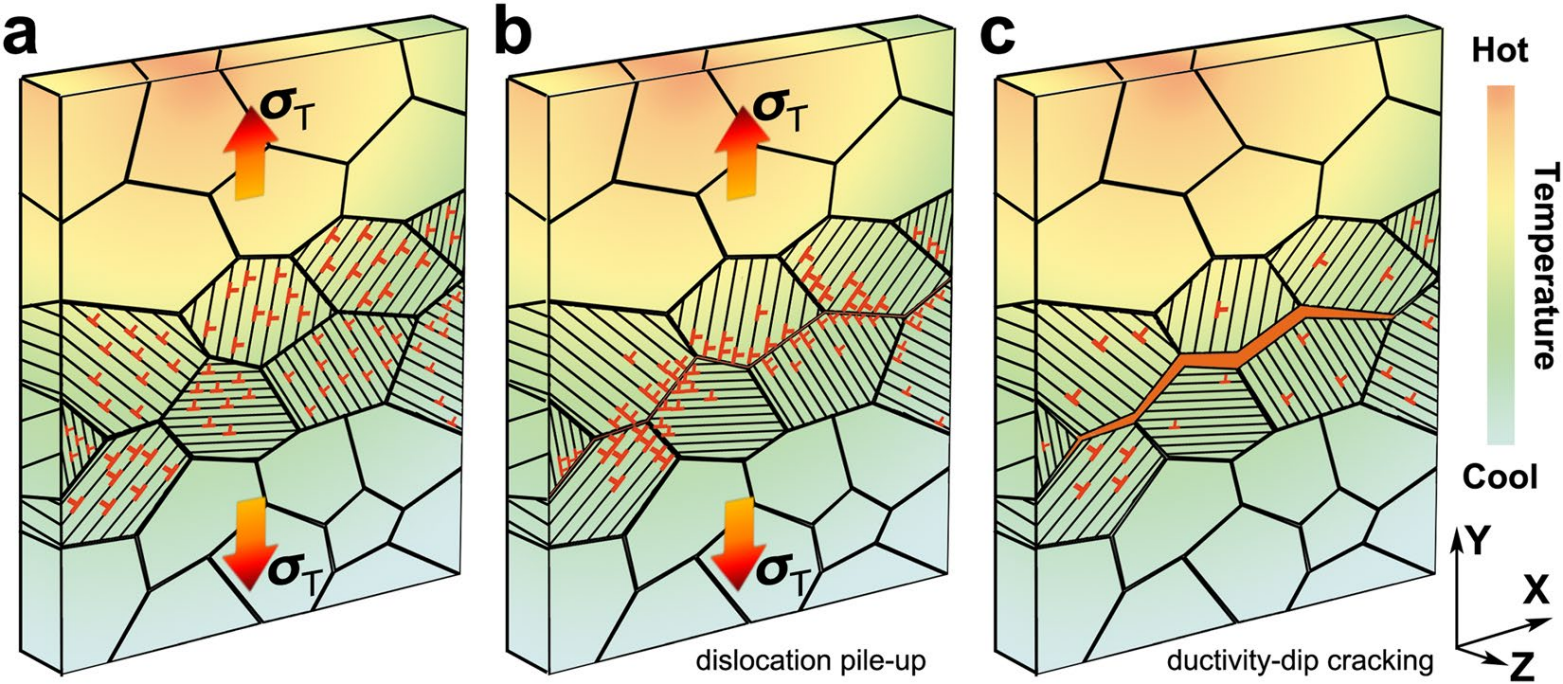
Possible schematic of the crack formation mechanism induced by thermal stress. (**a**) Sample under thermal stresses in the possible directions induced by temperature gradient, with dislocations activated mainly in the **XZ** plane. (**b**) Dislocations pile-up at the GBs, resulting in high deformation locally. (**c**) Void and crack formation caused by elevated local distortion.

From the experimental results, a qualitative explanation of dislocations activation, dislocation piled up, and crack formation at the GBs emerges and is demonstrated schematically in Fig. 6. First of all, it must be emphasized that the crack formation temperature has to be lower than the melting temperature, thus the crack forms after solidification. On the other hand, elevated temperature is still necessary to generate thermal gradient and thermal stress, and also reduce the material ductility. Once the tensile thermal stress, roughly perpendicular to the **XZ** plane, reaches the yield stress of the crystal grain, GNDs are activated, according to Schmid’s law. The dislocations slipped inside the crystal grains until they stopped and accumulated at HAGBs, where the materials microstructure becomes discontinuous. It should be mentioned that dislocations with opposite signs will propagate in the opposite direction until they meet the other side of the grain boundaries. However, according to our observation, the misorientation angles of the crystal grains adjacent to the crack are statistically higher than the average. It suggests that the dislocations are more probably blocked by the GBs with higher misorientation angles than the ones with lower misorientation angles. As the local density of dislocations at GBs increases, and perhaps with assistance from intergranular precipitates, the local strain near the GBs may exceed the material inherent ductility, resulting in void formation and eventually crack formation. From previous studies and postulates, medium homologous temperature favors crack formation, because in this temperature range the ductility of the materials is low. This statement is however difficult to be experimentally proven or rejected from our available data.

## Conclusion

In summary, an intergranular crack in the stray grain region of laser printed Ni-based DZ125L superalloy was measured with μXRD to study the microstructural evolution during DDC. The dislocation slip system in 40 crystal grains right next to the crack was investigated by analyzing the Laue diffraction peak shapes and then the equivalent uniaxial tensile force directions were estimated from Schmid’s law. Results showed that edge-type GNDs dominate in all these 40 grains in the ductility dip region and their dislocation line directions were almost parallel to the crack plane. The projected equivalent force directions distributed roughly normal to the average plane of the crack, suggesting that thermal stress played the key role to introduce the mechanical deformation of the materials here. Based on the experimental observations, a qualitative dislocations activation, dislocation piled-up, and crack formation mechanism has been proposed. Significant temperature gradient during laser 3D printing generates thermal stress within material to activate the dislocations. The dislocations slip inside the crystal grains but when they meet HAGBs they have a chance to be blocked. The local pile-up of dislocations will further enhance the strain/stress, until exceeding the inherent ductility of the materials at the GBs, favoring voids formation, growth, and coalescence into cracks.

## Methods

### Laser 3D printing

The laser direct forming was conducted using an independently developed system, XJTU-I^31^, equipped with a neodymium doped yttrium aluminum garnet (Nd:YAG) laser with beam size of 0.5 mm. Protected in a high purity argon (Ar) atmosphere, spherical powders with the diameter of 50–100 μm of Ni-based DZ125L superalloy were injected coaxially, with a feeding rate of 8 mm^3^/s, into the molten pool on the thin-walled directionally solidified DZ125L substrate. The molten pool was formed by laser heating with a power of 230 W. Then the laser beam scanned on the sample surface with a scanning speed of 8 mm/s, and the molten materials solidified and grew. The single-channel scanning process was repeated dozens of times with a **Y**-axis increment of 0.1 mm and thus a thin-walled part of layer-by-layer cladding was achieved. In the first few layers, columnar grains grew epitaxially with the substrate, and afterwards a columnar-to-equiaxed transition (CET) took place, and then fine grained polycrystals were obtained^25^. A crack containing specimen was cut from the polycrystalline region, mounted in epoxy, and then polished mechanically to obtain a mirror finish of the **XY** cross-sectional plane.

### μXRD study

μXRD measurement was carried out on Beamline 12.3.2 at the Advanced Light Source (ALS) of the Lawrence Berkeley National Laboratory (LBNL)^32^. In this technique, a high-brilliance synchrotron polychromatic X-ray beam (5–24 keV) was focused to a spot size of about 1 × 1 μm^2^ using a pair of Kirkpatrick-Baez mirrors. The laser 3D printed sample was mounted on a high resolution **X**-**Y** scanning stage and tilted 45° relative to the incident X-ray beam. The vertical **Y**-scanning direction corresponded to the cladding direction, and the horizontal **X**-direction corresponded to the laser scanning direction. A crack-containing area of 400 μm (horizontal) × 130 μm (vertical) was scanned with a scanning step size of 2 μm. Since the crystalline grain size in the Ni-based alloy was larger than the micro-focused X-ray beam, single crystal Laue diffraction patterns were generated at each scanning position, and recorded in reflection mode with a two-dimensional (2D) DECTRIS Pilatus-1M detector mounted at 90° with respect to the incoming X-ray, approximately 150 mm above the probed spot. At each scanning position the exposure time to obtain a diffraction pattern was 1 s. Diffraction peak positions were determined by fitting each reflection intensity profile with a 2D Gaussian function. The diffraction geometry, including the sample-to-detector distance, the center channel on the detector, and the relative tilts of the detector, was first calibrated by indexing a Laue pattern of a strain-free single crystal silicon chip. All the Laue patterns taken on the specimen were indexed using that same calibration. This approach secures high angular resolution (0.01°) for crystalline orientation^33, 34^, which is important for the evaluation of the quality of single crystals. Furthermore, by studying diffraction peak shapes, information on defects especially dislocations was also obtained^35, 36^, which provides essential clues to unveil the plastic deformation of metallic materials.

### Morphology characterization

After the μXRD experiment, the same laser direct formed specimen was etched using fresh nitro-hydrochloric acid for 5 s and then investigated under an optical microscope to obtain the metallurgical image of the identical crack-containing area. Another region containing a similar crack was polished to obtain a mirror finish of the **XY** cross-sectional plane, and deep Vickers indentations were made around this region as markers. After the surface topology of the marked region was recorded in a SU6600 field emission SEM, the specimen was polished again to further thin down by 10 μm, monitored using a micrometer. This process was repeated until the crack completely disappeared. Finally all the SEM images were added up sequentially using the software Mimics to obtain the 3D morphology of the crack. Another crack-containing specimen cut from the same laser cladding sample was carefully torn off to show the crack plane. The crack plane morphology was examined using a SU6600 field emission SEM, which was operated in secondary electron mode under the accelerating voltage of 15 kV.

## Electronic supplementary material

Supplementary Information
