# Supplementary material for: Statistical study of ductility-dip cracking induced plastic deformation in polycrystalline laser 3D printed Ni-based superalloy
Source: Sci Rep. 2017 Jun 6;7:2859. doi: 10.1038/s41598-017-03051-x (PMC5460225; doi:10.1038/s41598-017-03051-x)
Supplement: Supplementary file 1 — Supplementary Information [file 41598_2017_3051_MOESM1_ESM.pdf]

Statistical study of ductility-dip cracking induced plastic deformation in polycrystalline laser 3D-printed Ni-based superalloy

Dan Qian[1,a], Jiawei Xue[1,a], Anfeng Zhang[2], Yao Li[1,], Nobumichi Tamura[3], Zhongxiao Song[1], Kai Chen[1,*]

1. State Key Laboratory for Mechanical Behavior of Materials, Xi'an Jiaotong University, Xi'an, Shaanxi 710049, P.R. China
2. State Key Laboratory for Manufacturing Systems Engineering, Xi'an Jiaotong University, Xi'an, Shaanxi 710049, P.R. China
3. Advanced Light Source, Lawrence Berkeley National Laboratory, Berkeley, California 94720, USA

a. These authors contributed equally to this work

*Author to whom correspondence should be addressed. Email: kchenlbl@gmail.com

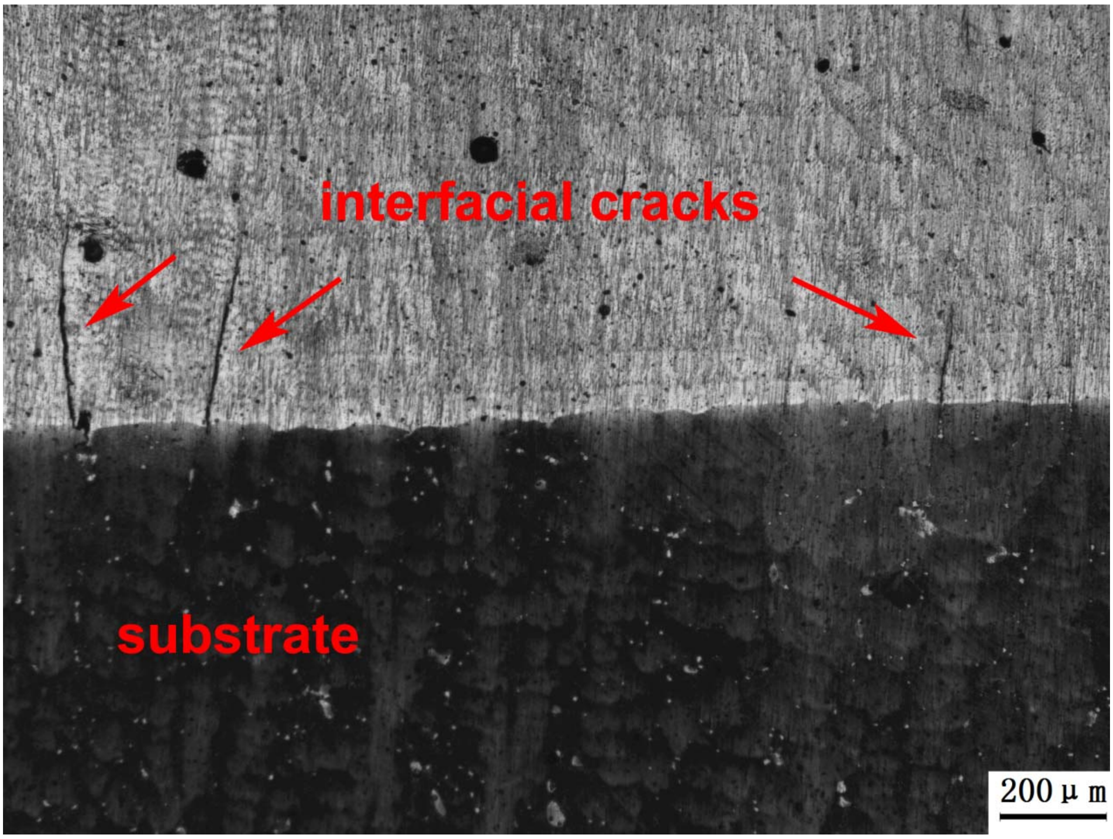

Figure S1 | Optical microscope image for cross-section of interfacial region, showing one cracking type in the deposited materials that the interfacial cracks can be visualized close to and perpendicular to the substrate-cladding interface on the cladding side.
